# Metabolome disruption of pregnant rats and their offspring resulting from repeated exposure to a pesticide mixture representative of environmental contamination in Brittany

**DOI:** 10.1371/journal.pone.0198448

**Published:** 2018-06-20

**Authors:** Nathalie Bonvallot, Cécile Canlet, Florence Blas-Y-Estrada, Roselyne Gautier, Marie Tremblay-Franco, Sylvie Chevolleau, Sylvaine Cordier, Jean-Pierre Cravedi

**Affiliations:** 1 Univ Rennes, EHESP, Inserm, Irset (Institut de recherche en santé, environnement et travail)—UMR_S 1085, Rennes, France; 2 INRA UMR 1331 Toxalim, University of Toulouse, INP, ENVT, EIP, UPS, UMR1331, Toulouse, France; Universiteit Gent, BELGIUM

## Abstract

The use of pesticides exposes humans to numerous harmful molecules. Exposure in early-life may be responsible for adverse effects in later life. This study aimed to assess the metabolic modifications induced in pregnant rats and their offspring by a pesticide mixture representative of human exposure. Ten pregnant rats were exposed to a mixture of eight pesticides: acetochlor (246 μg/kg bw/d) + bromoxynil (12 μg/kg bw/d) + carbofuran (22.5 μg/kg bw/d) + chlormequat (35 μg/kg bw/d) + ethephon (22.5 μg/kg bw/d) + fenpropimorph (15.5 μg/kg bw/d) + glyphosate (12 μg/kg bw/d) + imidacloprid (12.5 μg/kg bw/d) representing the main environmental pesticide exposure in Brittany (France) in 2004. Another group of 10 pregnant rats served as controls. Females were fed *ad libitum* from early pregnancy, which is from gestational day (GD) 4 to GD 21. Urine samples were collected at GD 15. At the end of the exposure, mothers and pups were euthanized and blood, liver, and brain samples collected. ^1^H NMR-based metabolomics and GC-FID analyses were performed and PCA and PLS-DA used to discriminate between control and exposed groups. Metabolites for which the levels were significantly modified were then identified using the Kruskal-Wallis test, and p-values were adjusted for multiple testing correction using the False Discovery Rate. The metabolomics analysis revealed many differences between dams of the two groups, especially in the plasma, liver and brain. The modified metabolites are involved in TCA cycle, energy production and storage, lipid and carbohydrate metabolism, and amino-acid metabolism. These modifications suggest that the pesticide mixture may induce oxidative stress associated with mitochondrial dysfunction and the impairment of glucose and lipid metabolism. These observations may reflect liver dysfunction with increased relative liver weight and total lipid content. Similar findings were observed for glucose and energy metabolism in the liver of the offspring, and oxidative stress was also suggested in the brains of male offspring.

## Introduction

Pesticides are commonly used in agriculture. Their application can lead to environmental contamination. Although human exposure is not fully elucidated, numerous studies have shown that proximity to areas of agricultural pesticide use may be a source of pesticide exposure, in addition to domestic or dietary sources [1–5]. Exposure to pesticides is hazardous to human health, particularly in occupational situations, in which increased risks of cancer, developmental defects, or neurologic problems have been observed [6,7]. Such conclusions are more difficult to draw for low-dose environmental exposure to pesticides. However, fetuses and infants are particularly susceptible to toxicants. An analysis of published studies shows that prenatal exposure to some pesticides may induce malformations (herbicides) [8–10], affect fetal growth (herbicides, organophosphorous) [11–13], or are associated to behavioral disorders (organophosorous, pyrethroids) [14–23].

Metabolomics is a promising approach to study the associations between environmental exposures and health effects. Over the last few years, many toxicological studies have demonstrated that metabolomics is a powerful method for detecting changes in the metabolome of individuals exposed to pesticides. A recent comprehensive review showed that exposure to pesticides mixtures may induce metabolic modifications potentially linked with physiopathological disturbances [24]. Endosulfan, atrazine, and chlorpyrifos in combination can potentially induce changes in amino-acid metabolism, the citrate cycle, the urea cycle, and glucose metabolism through an oxidative disturbance in mice [25,26]. Exposure to organophosphorus mixtures (chlorpyrifos and carbaryl, or dichlorvos, dimethoate, acephate, phorate) induces disturbances in energy and lipid metabolism in rats [27–29]. Altered lipid metabolism has also been recently confirmed for exposure to organophosphorus.: hepatic dysfunction, such as non-alcoholic fatty liver disease, has been observed in rats exposed to dichlorvos + acephate + phorate + dimethoate [30]. Energy metabolism has also been shown to be altered by exposure to pyrethroids (deltamethrin) in combination with dichlorvos [31]. Toxicological studies are usually carried out in conditions which are not representative of human exposures, in particular because they investigate simple mixtures at high doses. Only one study has been designed to investigate the metabolic effects of complex mixtures of pesticides at lower doses: Mehri *et al*. investigated the effect in mice of a mixture of low-doses (calculated from their respective acceptable daily intakes defined by the joint FAO/WHO meeting on pesticide residues and extrapolated to the mice on the basis of mean body weight values) of six pesticides frequently found in fruits and vegetables grown in France and showed a metabolic signature linked to oxidative stress and glucose regulation [32].

More than 60% of the surface area in Brittany (France) is devoted to agricultural activities, with 50% devoted to cereals and corn. In the 2000’s, almost all land in this region received at least four different treatments to control the proliferation of annual grasses, fungi, and insects. In 2004, acetochlor (chloroacetanilide herbicide), carbofuran (carbamate insecticide), chlormequat (quaternary ammonium plant growth regulator), ethephon (organophosphorus plant growth regulator), fenpropimorph (morpholine fungicide), imidacloprid (neonicotinoid insecticide), glyphosate (glycine derivative herbicide), and bromoxynil (nitrile herbicide) constituted almost 90% of the pesticides used on cereals and corn. A previous study based on 83 pregnant women from the PELAGIE cohort showed changes in the urinary metabolome of women living in towns with high agricultural cereal activities [33]. These changes are potentially related to energy metabolism. The objective of the present study was to examine the effects in pregnant rats of a realistic pesticide mixture based on substances applied to crops in Brittany to support these previous observations.

## Material and methods

### Chemicals and exposure

Methanol (purity ≥ 99.8) was purchased from Merck (Darmstadt, Germany). Ultrapure water was produced with a Milli-Q system (Millipore, Saint Quentin en Yvelines, France) and was used for pesticide dissolution. The eight high-purity (≥ 98%) pesticides (pestanal^®^ analytical standards) acetochlor (CAS 34256-82-1, Batch SZB9314XV), bromoxynil (CAS 1689-84-5, Batch SZB8021XV), carbofuran (CAS 1563-66-2, Batch SZB9064XV), chlormequat (CAS 999-81-5, Batch SZB8248XV), ethephon (CAS 16672-87-0, Batch SZBB021XV), fenpropimorph (CAS 67564-91-4, Batch SZB9243XV), glyphosate (CAS 1071-83-6, Batch SZB9320XV), and imidacloprid (CAS 138261-41-3, Batch SZB9112XV), were purchased from Fluka, Sigma Aldrich (Les Ulis, France). They were incorporated into the rat diet, at a nominal dose corresponding to the same proportion as their respective environmental exposure based on French use in 2004 (https://www.airbreizh.asso.fr/), to reach a total dose of 447 μg/kg bw/d, which corresponds to the sum of their respective acceptable daily intake. Theoretical and ingested doses calculated from food consumption during the experiment are shown in Table 1.

**Table 1:** Ingested doses of pesticides by dams (μg/kg bw/day), calculated from a total dose based on the sum of the acceptable daily intake (ADI) of each pesticide and the proportion to their level of environmental exposure in Brittany (France).

Pesticide: Name (chemical class)	Theoretical oral dose	Dietary intake based on food consumption	ADI^a^ (POD^b^ used for its derivation) in μg/kg bw/day	Proportion of environmental pesticide exposure in Brittany (%)^c^
***Acetochlor (chloroacetanilide)***	*290*	***246***	*3*.*6 (LOAEL*: *1080)*	*64*.*83*
***Bromoxynil (nitrile)***	*14*	***12*.*0***	*10 (NOAEL*: *1000)*	*3*.*16*
***Carbofuran (carbamate)***	*27*	***22*.*5***	*0*.*15 (LOAEL*: *30)*	*6*.*03*
***Chlormequat (quaternary ammonium)***	*42*	***35*.*0***	*40 (NOAEL*: *4000)*	*9*.*33*
***Ethephon (organo-phosphorus)***	*27*	***22*.*5***	*30 (NOAEL*: *27000)*	*6*.*03*
***Fenpropimorph (morpholine)***	*18*	***15*.*5***	*3 (NOAEL*: *300)*	*4*.*02*
***Glyphosate (glycine derivative)***	*14*	***12*.*0***	*300 (NOAEL*: *31000)*	*3*.*17*
***Imidacloprid (neonicotinoid)***	*15*	***12*.*5***	*60 (NOAEL*: *3700)*	*3*.*43*

^a^ acceptable daily intake from EFSA and Agritox, data from the Decision of the European Union, available at www.efsa.europa.eu and www.agritox.anses.fr, respectively;

^b^ POD: point of departure (NOAEL: no observed adverse effect level, or LOAEL: lowest observed adverse effect level);

^c^ the proportions of pesticide used in this study were defined from a registry of pesticide’s emissions achieved in 2003 in Brittany (France) by a regional association for air quality https://www.airbreizh.asso.fr/).

This registry was based on different practice’s surveys implemented on the water catchment scale, the physical chemical characteristics of the pesticides, and the spreading quantities at the square-kilometer scale.

### Preparation of the contaminated diet

Commercial powdered diet formulated for growth, pregnancy, and lactation (A03, SAFE) was used for this study. We first incorporated the pesticides into a premix batch, because of the extremely low quantities added to the diet, by thoroughly mixing the required quantity of each pesticide with 50 g powdered diet. Briefly, all pesticides were dissolved in methanol (10 mL total volume), except glyphosate, which was dissolved in water (1 mL), and both alcoholic and aqueous solutions were added to the diet. The mixture was then homogenized for 15 min using a Virtis 45 Blade-type homogenizer (Gardiner, New York). The resulting mix was kept for 24 h at room temperature to allow the methanol and water to evaporate. The mix was then combined with 50 g diet and put in a Dangoumau ball-mill (Prolabo, Fontenay-sous-Bois, France) to be reduced to a powder. This premix was stored at 4°C in glass flasks until mixing and pelleting of the diet. The same procedure was used for the control diet, except that no pesticide was added to the alcoholic and aqueous solutions. Before pelleting, all diet ingredients and the premix were mixed for approximately 20 min in a mixing bowl (Santos, Asmo Sud, Toulouse, France).

### Animals

Twenty female Wistar Crl:WI(Han) rats were purchased from Charles River Laboratories, L’Arbresle, France. Rats were mated the day before receiving the diet, considered to be gestational day (GD) 1. Animals were acclimated for two days before exposure. Animals were maintained under controlled temperature and light (21°C ± 2°C, 12-hours light/dark cycle). The mean body weight was 212 g at the beginning of the experiment. Females (10 per lot) were fed control or contaminated diets from GD4 to GD21. Food consumption and body weight were measured every two days. At GD13, eight females per group were placed in metabolic cages and acclimated for two days before a 24-h urine collection on GD15 (the end of the organogenesis). At GD21, approximately 12 h before the expected time of parturition, animals were sacrificed by cervical dislocation followed by exsanguination. Blood samples were taken from the facial artery of the dams, added to a glass vial containing heparin, and placed on ice before centrifugation and freezing at -20°C. At the same time, the fetuses were withdrawn from the uterus of the dams for blood recovery. For each individual, the liver (the median lobe for dams, and a pool of three entire livers from each litter for male and female fetuses) and the brain (entire for dams, and a pool of three entire brains from each litter for male and female fetuses) were excised, weighed, and rapidly frozen in liquid nitrogen.

The study was conducted in accredited animal care facilities (#C31 555 13) by an approved staff, and animal care was in accordance with the guidelines of the European Council on Animals used in Experimental Studies. All experimental protocols and procedures were approved by the local Institutional Animal Care and Use Committees from INRA: "Comité d'Ethique de Pharmacologie-Toxicologie de Toulouse Midi-Pyrénées" (CEEA#86 / Ministry of Higher Education, Research and Innovation) (TOXCOM/0124/JPC, national number APAFiS#4303_2016022912425905_V3), according to the Directive 2010/63/EU recommendations.

### Metabolomics analyses

#### Sample preparation

Urine samples: After thawing at room temperature and vortexing, 500 μL urine was mixed with 200 μL phosphate buffer (pH 7.39) prepared in D_2_O to which was added sodium 3-trimethylsilyl-1-[2,2,3,3,-^2^H_4_]-propionate (TSP, 1 mM). The phosphate buffer is used to minimize variations in chemical shift values in the acquired NMR spectra due to pH differences. TSP served as a chemical shift reference (δ 0 ppm) and D_2_O as a field-frequency lock for the NMR spectrometer. Each sample was vortexed and centrifuged for 10 min at 6,080 g to remove any precipitate. Then, 600 μL aliquots were transferred to standard 5 mm—NMR tubes for analysis.

Plasma samples: After thawing at room temperature and vortexing, 200 μL serum was mixed with 500 μL D_2_O. Each sample was vortexed and centrifuged for 10 min at 6,080 g and 600 μL aliquots were transferred to standard 5 mm—NMR tubes for analysis. Liver and brain samples: Extraction procedures were derived from the method described by Waters *et al*. [34].

Tissue samples: Liver (100 mg) and whole brain were homogenized using a Polytron PT2100 in acetonitrile/ H_2_O (50/50, v/v) containing 0.1% BHT in an ice-water bath. Homogenates were centrifuged at 5,000 g for 10 min at 4°C and the supernatants removed and lyophilized before being reconstituted in 600 μL D_2_O containing 0.25 mM TSP (as a chemical shift reference at 0 ppm).

#### ^1^H NMR spectra acquisition

All ^1^H NMR spectra were acquired at 300 K on a Bruker Avance DRX-600, operating at 600.13 MHz (Bruker Biospin, Wissembourg, Germany), equipped with an autosampler and an inverse ^1^H-^13^C-^15^N cryoprobe. For urine samples, ^1^H NMR spectra were acquired using a standard pulse sequence NOESY to suppress water resonance. A relaxation delay of 2 s and mixing time of 150 ms were used and 256 free induction decays (FIDs) were collected into 32 k data points using a spectral width of 20 ppm with an acquisition time of 1.36 s. ^1^H NMR spectra were acquired using the Carr-Purcell-Meiboom-Gill (CPMG) spin-echo pulse sequence with pre-saturation, with a total spin-echo delay (2 nt) of 240 ms to attenuate broad signals from proteins and lipoproteins. A total of 128 transients were collected into 32 k data points using a spectral width of 20 ppm, a relaxation delay of 2 s and an acquisition time of 1.36 s.

#### ^1^H NMR data preprocessing

All free induction decays were then multiplied by an exponential function with a line broadening factor of 0.3 Hz prior to Fourier transformation. All spectra were referenced to the chemical shift of TSP (δ 0.00). All NMR spectra were manually phase- and baseline-corrected using Topspin (V2.1, Bruker Biospin, Germany) and ACD/NMR Processor (Academic Edition, ACD Labs, Canada). The spectral regions containing residual water, solvents, and urea resonances were removed and the spectra digitized to 469 to 766 buckets (according to the biological media) corresponding to 0.01 ppm intervals using the AMIX software package (V3.9.11, Bruker Biospin, Germany). Each integrated region was divided by the total spectral intensity to normalize values. This partially removes concentration differences between samples.

#### GC-FID analysis of fatty acid methyl esters in the liver

Tissue samples were homogenized using the Fastprep system (40 s) in methanol/water (83/17 v/v). Dichloromethane (2 mL) was added to the homogenates and, after vortexing, a dichloromethane/water (50/50 v/v) mixture (4 mL) was added. After 15 min at 4°C, the extracts were centrifuged (2,870 g for 15 min at 4°C), and the lower lipophilic phase recovered and evaporated to dryness under a nitrogen stream. The liver samples from adult females and all offspring were retaken by 5 mL and 1 mL of dichloromethane, respectively. An aliquot corresponding to 5 mg of tissue was sampled and 5 μg TG17 (glyceryl triheptadecanoate, Sigma Aldrich, les Ulis, France) added as internal standard to verify the completeness of hydrolysis. After hydrolysis in KOH-methanol (0.5 M) for 30 min at 56°C, FAs were transmethylated with 1 mL BF_3_-methanol, 10% wt (60 min at 80°C). Once cooled down, 1 mL Milli-Q water and 2 mL heptane was added to the methylated FAs and the mixture manually shaken. After centrifugation (500 g, 5 min), the upper layer containing FA methyl esters (FAMEs) was transferred to a glass tube and evaporated to dryness. Heptane (200 μL) was then added and the sample transferred to a vial. FAMEs were analyzed on a TRACE 1310 gas chromatograph (Thermo Scientific, Les Ulis, France) equipped with a split-splitless injector operated in the splitless mode and a flame-ionization detector. FAMEs were separated on a FAMEWAX^TM^ column (30 m, 0.32 mm internal diameter, 0.25 μm film thickness) from Restek (Lisses, France) using helium as carrier gas at a constant flow of 1.0 mL.min^-1^. The injector temperature was set at 225°C and the oven temperature was programmed as follows: 1 min isothermal step at 130°C, from 130°C to 245°C at 2°C.min^-1^ and then 8 min at 245°C. FAMEs were identified by comparing sample retention times to those of commercial standard mixtures (Menhaden oil and Food Industry FAME Mix, Restek) using Xcalibur 2.2 software (Thermo Scientific).

#### Discriminant analysis according to exposure groups

The NMR spectral and GC-FID data integration were imported into the SIMCA-P+ software package (version 12.0, Umetrics) for multivariate statistical analysis. Principal component analyses (PCA) was performed to separate the exposed and control groups and remove outliers, if necessary. The PLS-DA method was then applied to identify potential metabolites corresponding to the buckets with variable importance in the projection (VIP) above 1. Cross-validation was used to determine the number of linear combinations to be included in the PLS-DA model. The quality of the model was given by the two parameters, R2Y (proportion of explained variance) and Q2Y (predictive ability). The Q2 value was evaluated using a seven-fold cross-validation. A permutation test (200 iterations) was conducted for each PLS-DA model to test their validity. If no separation was obtained with preliminary PCA or PLS-DA, we used orthogonal signal correction (OSC) filtering to decrease variability in the X-matrix (spectral data) not correlated with the exposure groups [35,36], which include confounding factors such as physiological, experimental, and instrumental factors.

A non-parametric Kruskal-Wallis test with a critical p-value of 0.05 was further used to determine whether there was a significant difference between the two groups for each metabolite obtained from the PLS-DA models. P-values were adjusted for multiple testing correction using the False Discovery Rate [37]. This test was conducted using R software (version 2.12.1). Statistically significant changes between the lipid content of liver and brain from the exposed and control groups were also assessed using a non-parametric Kruskal-Wallis test (n = 6 to 10 per group, p-value < 0.05).

#### Metabolite identification

Spectral assignments were based on matching 1D NMR data to reference spectra in a home-made reference database, as well as with other databases (http://www.bmrb.wisc.edu/metabolomics/; http://www.hmdb.ca/), and reports in the literature.

## Results

Characteristics of the animals are presented in Table 2. Our results do not show any significant differences in body weight, body weight gain, or absolute liver weight in exposed dams relative to controls. Food consumption was slightly but significantly increased in exposed dams in comparison to control dams, but the difference disappeared after adjusting for body weight. The relative liver weights of exposed dams were slightly but significantly reduced than those of the control dams, and this was associated with a significant increase in lipid mass per gram of liver. The brains of exposed dams were significantly heavier than those of the exposed group (p < 0.05), but the difference disappeared after adjusting for body weight. Dams exposed to pesticides had fewer female fetuses than non-exposed dams, but the number of offspring per litter was the same. Furthermore, the mean liver weight of male fetuses in the exposed group was significantly higher than that of those of the non-exposed group (p < 0.005), whereas the mean brain weight of fetuses of both genders was higher (p < 0.01 for males, p < 0.05 for females). This suggests possible gender-specific sensitivity to this pesticide mixture, with males possibly being more sensitive than females.

**Table 2:** Food consumption and body and organ weights for dams and offspring.

Parameters	Control group	Exposed group
***Dams***	*(n = 10)*	*(n = 10)*
Food consumption (g)	294.3 ± 18.3^a^	317.2 ± 31.8
Relative food consumption/body weight (%)	1.43 ± 0.10	1.47 ± 0.13
Body weight gain (g)	97.5 ± 6.3	95.5 ± 13.3
Body weight at GD21 (g)	302.7 ± 12.6	312.2 ± 22.5
Absolute liver weight (g)	9.49 ± 0.82	8.98 ± 0.98
Relative liver/ body weight (%)	**3.13 ± 0.19**	2.87 ± 0.19*^b^
Lipid mass per gram of liver (mg/g)	**26.20 ± 4.52**	33.77 ± 5.50**
Brain weight (g)	**1.44 ± 0.18**	1.57 ± 0.07*
Relative brain/ body weight (%)	0.48 ± 0.06	0.50 ±0.03
***Offspring***		
Number of offspring per litter	11.39 ± 1.6	10.28 ± 1.6
Number of males	5.71 ± 1.03	5.47 ± 2.13
Number of females	**5.53 ± 1.34**	4.20 ± 1.51*
Liver weight ^c^ (g)		
Males	**0.45 ± 0.08**	0.58 ± 0.10***
Females	0.56 ± 0.11	0.60 ± 0.08
Lipid mass per gram of liver (mg/g)		
Males	**20.09 ± 3.92**	16.1 ± 3.56*
Females	15.73 **±** 3.42	15.18 **±** 2.17
Brain weight ^c^ (g)		
Males	**0.36 ± 0.05**	0.42 ± 0.04**
Females	**0.39 ± 0.02**	0.41 ± 0.03*

^a^ Data shown as the mean ± SD.

^b^ Comparison with the Kruskal Wallis test:

*p < 0.05;

**p < 0.01;

***p < 0.005.

^c^ pool of three individuals per litter.

### Comparison of metabolic profiles of exposed dams versus control dams

We compared the ^1^H NMR spectra of urine (at GD15), plasma (at GD21), and liver and brain extracts of exposed and control dams (raw data are available in S1–S4 Tables): the fingerprints resulting from dietary exposure to the pesticide mixture were comprised of 35 metabolites.

#### Urinary metabolic profiles

**Fig 1:**
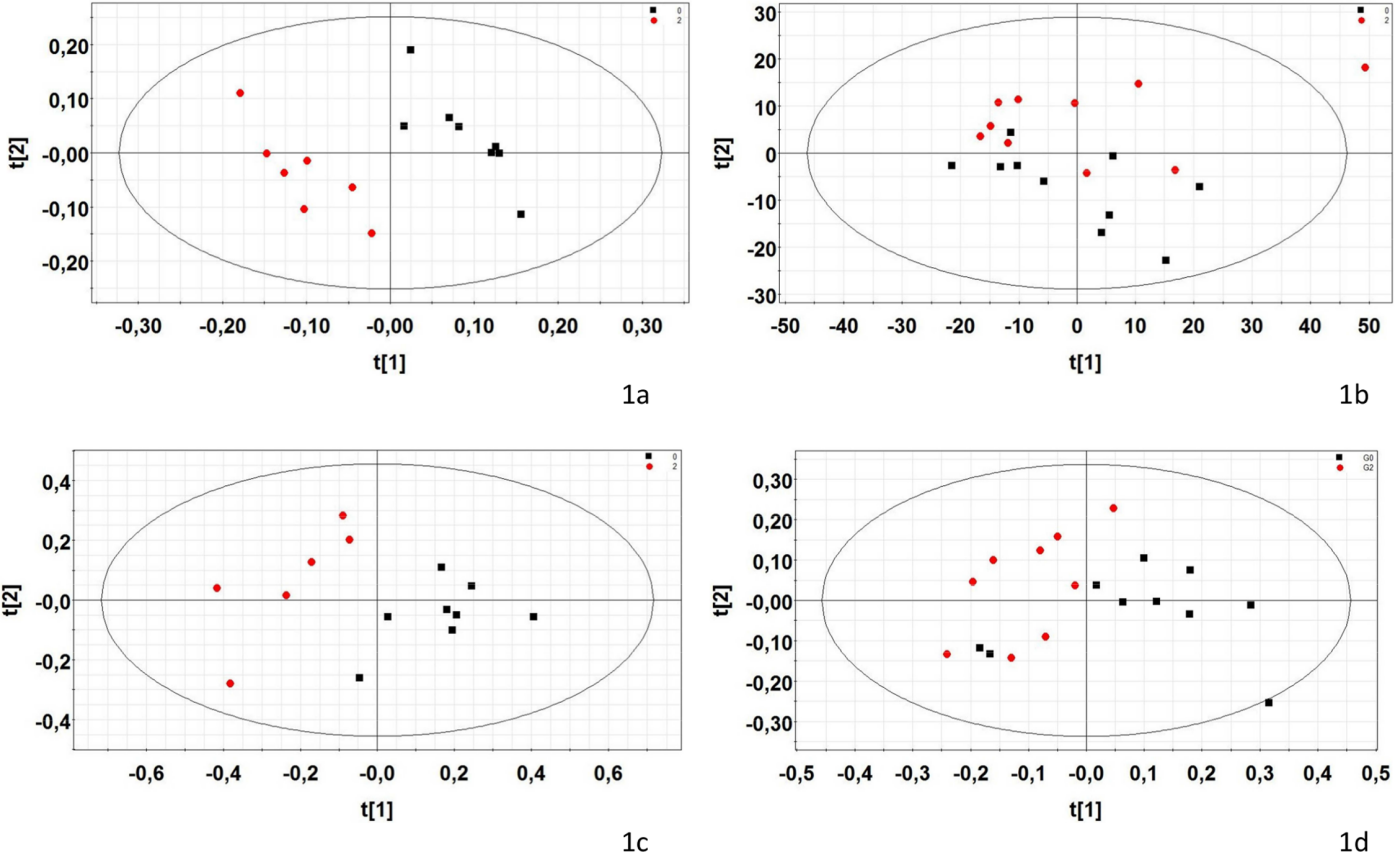
Two-dimensional PLS-DA and PCA scores plot of pregnant rat samples integrated ^1^H NMR spectra. 1a: GD 15 urine samples (PLS-DA, A = 2, R2 = 94.8%, Q2 = 0.613); 1b: GD 21 plasma samples (PCA, A = 4, R2 = 72.5%); 1c: GD21 liver aqueous extract samples (PCA, A = 3, R2 = 61.6%); 1d: GD21 brain aqueous extract samples (PCA, A = 4, R2 = 72.8%). Each dot represents an observation (animal), projected onto first (horizontal axis) and second (vertical axis) PLS-DA or PCA variables. Control group is shown with black squares and the group exposed to eight pesticides widely used in Brittany (France) in 2004 (acetochlor, bromoxynil, carbofuran, chlormequat, ethephon, fenpropimorph, glyphosate, imidacloprid) is shown with red circles. The black ellipse determines the 95% confidence interval, which is drawn using Hotelling’s T2 statistic.

The first PCA of urine metabolites revealed a chemical shift for citrate, possibly due to a different urinary pH between individuals, despite the addition of phosphate buffer. After excluding the spectral regions corresponding to the signals of the citrate protons (δ 2.51–2.58 ppm and δ 2.65–2.73 ppm), neither preliminary PCA nor PLS-DA separated the exposed group from the controls. An OSC filtered PLS-DA model allowed us to identify two metabolites (hippurate, citrulline) for which significant differences in the signal were confirmed using the Kruskal-Wallis test. Differences were also shown for three other metabolites (creatine, phenylacetylglycine and taurine) without statistical significance (0.05<p<0.08). Characteristics of the modelling are presented in the Table 3 and score plots in the Fig 1A. Additionally, a specific analysis of the spectral regions corresponding to the citrate using the Kruskal-Wallis test shows a statistical significant increase in the exposed group compared to the control group (p = 0,001).

**Table 3:** Results of the partial least square modelling of metabolic profiles of dams (urine, plasma, aqueous liver and brain tissue extracts, and lipidic liver tissue extracts).

Samples	Number of rats	Model used	Number of LV^a^	R2	Q2	Intercepts^b^	Number of VIP^C^ > 1
**Urine**	15	OSC-pareto scaled + PLS-DA (mean centered)^d^	2	94.8%	0.613	R2 = (0.0, 0.665) Q2 = (0.0, -0.249)	163
**Plasma**	20	PLS-DA (pareto scaled)	3	80.6%	0.413	R2 = (0.0, -0.472) Q2 = (0.0, -0.232)	155
**Liver–aqueous**	14	PLS-DA (pareto scaled)^e^	1	81.2%	0.704	R2 = (0.0, -0.277) Q2 = (0.0, -0.222)	87
**Liver–Fatty acids**	20	OSC-unit scaled + PLS-DA (unit scaled)	1	93.3%	0.835	R2 = (0.0, 0.291) Q2 = (0.0, -0.166)	7
**Brain–aqueous**	19	PLS-DA (pareto scaled)^f^	2	86.5%	0.552	R2 = (0.0, 0.514) Q2 = (0.0, -0.231)	165

^a^ LV: latent variables.

^b^ 200 permutations.

^c^ VIP: variable importance in projection.

^d^ exclusion of the citrate region (shift) and rat n°G2-18 (outlier in a preliminary PCA).

^e^ exclusion of rats n°G2-17 and G0Mbis.

^f ^exclusion of rat n°G2-Mter.

#### Plasma metabolic profiles

Preliminary PCA revealed acceptable separation, especially on the second component (Fig 1B). Rat n° 23 was more distant than the other individuals, because of increased lipid and lactate content. PLS-DA modelling allowed us to identify 11 significantly altered metabolite signals for the exposed group relative to controls (higher for lipids, aceto-acetate, glutamate and dimethylamine and lower for creatine, acetate, alanine, glutamine, isoleucine, lysine and valine), suggesting a disturbance of energy, amino-acid, and lipid metabolism (Tables 3 and 4).

**Table 4:** Plasma, liver and brain metabolite levels that were significantly different between dams from the exposed and control groups.

	Plasma	Liver aqueous	Brain aqueous	Biological role
Hippurate				Gut microbial metabolism?
Citrulline				Urea cycle
Creatine	↘	↗ ^c^	↗^a^	Amino-acid synthesis, storage energy (phosphocreatine)
Phenylacetylglycine				Minor metabolites of fatty acids
Citrate				TCA cycle
Succinate			↘ ^b^	TCA cycle
ATP			↘	Energy source
ADP/AMP			↘	Energy source
Glycerol		↘		Lipid component, converted to glucose for energy production
Glycogen		↘		Energy storage
Glucose		↗		Energy source
Lactate			↘	Energy metabolism
Alanine	↘			Energy source, glucose metabolism regulator
Glutamate	↗	↘^b^		Neoglucogenesis, excitatory neurotransmitter
Glutamine	↘	↘	↘	Non-essential amino-acid, role in TCA cycle
Valine	↘			Essential amino-acid involved in stress, energy and muscle metabolism, role in carbohydrate synthesis
Acetate (with lysine)	↘	↗ ^c^		Lipid and carbohydrate metabolism
Lipids	↗		↘	Lipid metabolism
Aceto-acetate	↗			Lipid metabolism, cholesterol synthesis
3-hydroxybutyrate		↗ ^c^		Lipid metabolism. Energy source
Isoleucine	↘			““
Lysine (with acetate)	↘		↗	Essential amino-acid involved in stress, precursor of acetyl-coA
Serine		↘	↘	Non-essential amino-acid derived from glycine
Dimethylamine	↗			Gut microbial metabolism of choline, host co-metabolism
Dimethylgylcine		↘		Byproduct of homocysteine and glycine metabolism
Inosine			↗	Purine metabolism
Aspartate			↘	Non-essential amino-acid produced from glutamate, neurotransmitter.
N-acetylaspartate			↗	Neuronal osmolyte, lipid synthesis, derived from aspartate in brain
Oxidized gluthatione			↗	Anti-oxidant
Glycine		↘		Osmoprotector, defense mechanisms
Taurine		↘	↗^c^	Membranes stabilizer in brain, antioxidant, osmolyte
Glycero-phosphocholine		↗	↘	Membrane stabilizer, osmolyte
Phosphocholine			↘	Membranes stabilizer, osmolyte
Ethanolamine			↗	Membrane phospholipid synthesis
Uridine			↘	Nucleoside, synthesis of RNA membrane, regulation of physiological processes

^a^ with creatinine.

^b^ close to the signal of glutamine.

^c^ tendency but without statistical significance (0.05 < p < 0.08).

#### Liver metabolic profiles

Aqueous extract: After excluding two individuals with a high content of 3-hydroxybutyrate and highly diluted spectra, the PCA showed very good separation on the first component that explained 44% of the total variability between groups (Fig 1C). The first component of PLS-DA model explained 81.2% of the difference (Table 3). There was a significantly higher signal for glucose and glycerophosphocholine for the exposed group and to a lesser extent (tendency without significance), for creatine, acetate and 3-hydroxybutyrate. A concomitant decrease in the signal for glutamate, glutamine, glycogen, glycerol, serine, dimethylglycine, glycine, and taurine suggests an impact on energy, glucose, and amino-acid metabolism and potential oxidative stress (Table 4).

Lipid fraction: We undertook specific analyses of hepatic fatty acids because of the significant decrease in relative liver weight and increase in lipid content in the livers of exposed dams (see Table 2) and because of the relevance of the liver as a target organ for pesticides [38]. There was no statistically significant difference in the saturated/unsaturated fatty acid ratio between the exposed group and controls. Neither PCA nor PLS-DA separated the exposed and control groups. An OSC filtered PLS-DA model allowed us to identify seven fatty acids for which significant differences in the signal were confirmed using a Kruskal-Wallis test. The first component of the PLS-DA model explained 93.3% of the variability (Table 3). The signals for unsaturated fatty acids (PUFAs C20:2ω6, C20:3ω6, C20:4ω6; C22:4ω6, C22:5ω3, and MUFA C18:1ω7) and one saturated fatty acid (C18:0) were higher for the exposed group (Table 5), whereas those for C14:0, C16:0, C16:1ω7, C18:1ω9, C20:1ω9, C18:2ω6, C18:3ω6, C18:3ω3, C20:3ω9, C20:5ω3, C22:5ω6, C22:6ω3 were unaffected.

**Table 5:** Liver fatty acids for which the signals were significantly different between dams from the exposed and control groups (using the Kruskal Wallis test, p-value<0.05).

Fatty acids	Variables	Tendancy
**Arachidonic acid**	OSC:C20:4ω6	↗
**Eicosadienoic acid**	OSC:C20:2ω6	↗
**Dihomo-γ-linolenic acid**	OSC:C20:3ω6	↗
**Adrenic acid**	OSC:C22:4ω6	↗
**Stearic acid**	OSC:C18:0	↗
**Docosapentaenoic acid**	OSC:C22:5ω3	↗
**Vaccenic acid**	OSC:C18:1ω7	↗

#### Brain metabolic profiles (aqueous extracts)

After excluding one outlier (with a very concentrated spectrum which contains methanol, probably due to partial evaporation), the PCA properly separated the exposed group from the controls, especially on the first component (Fig 1D). PLS-DA modelling followed by a Kruskal-Wallis test allowed us to identify significantly higher signals for N-acetylaspartate, lysine, inosine, oxidized glutathione and ethanolamine in brain, and lower signals for ATP, ADP/AMP, lipids, glutamine, aspartate, succinate, lactate, serine, glycerophosphocholine, phosphocholine, and uridine. Non-significant increases in creatine and taurine were also observed (Table 4). These differences could be linked to oxidative stress.

### Comparison of the metabolic profiles at GD21 between exposed and control groups of offspring

#### Plasma metabolic profiles

**Fig 2:**
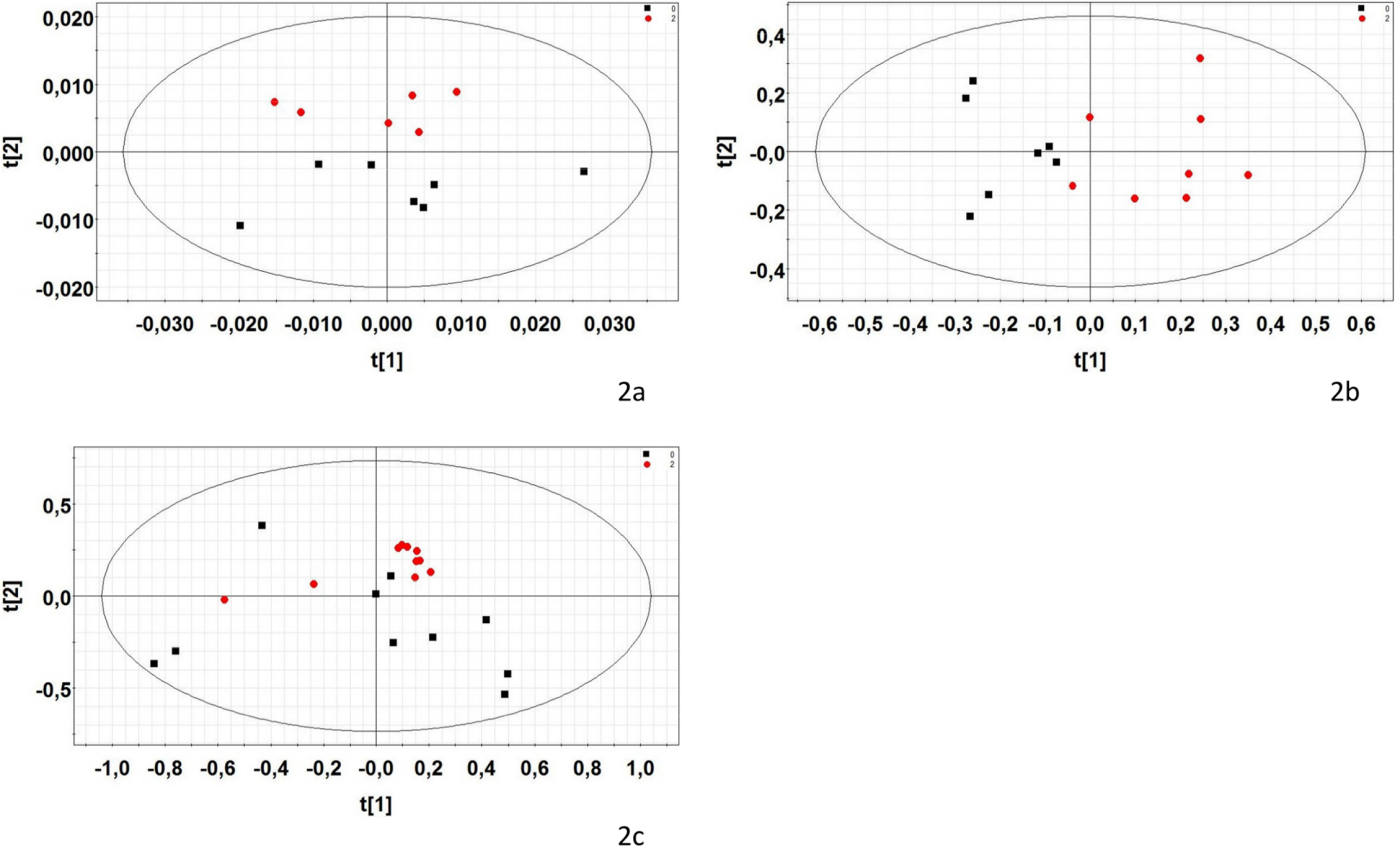
Two-dimensional PCA scores plot of offspring rat samples integrated ^1^H NMR spectra. 2a: GD21 plasma samples (males only) (PCA, A = 3, R2 = 87.9%); 2b: GD21 aqueous liver extracts of males samples (PCA, A = 4, R2 = 76.7%); 2c: GD21 aqueous liver extracts of females samples (PCA, A = 4, R2 = 87.1%). Each dot represents an observation (animal), projected onto first (horizontal axis) and second (vertical axis) PCA variables. Control group is shown with black squares and the group exposed to eight pesticides widely used in Brittany (France) in 2004 (acetochlor, bromoxynil, carbofuran, chlormequat, ethephon, fenpropimorph, glyphosate, imidacloprid) is shown with red circles. The black ellipse determines the 95% confidence interval, which is drawn using Hotelling’s T2 statistic.

Only male blood profiles were analyzed due to an insufficient quantity of blood collected from the female fetuses. Each NMR spectrum was acquired from pooled blood from three males per litter. Raw data are presented as bucket table in S5 Table. The preliminary PCA (mean-centered data) revealed good separation, especially on the second component (Fig 2A). PLS-DA modelling (characteristics given in Table 6) allowed to identify significant lower plasma level of glutamate and choline, and a higher plasma level of lipids in exposed male fetuses than controls (Table 7).

**Table 6:** Results of the partial least square modelling of metabolic profiles of offspring (plasma, liver, and brain tissue extracts).

Samples	Number of rats^a^	Model used	Number of LV^b^	R2	Q2	Intercepts^c^	Number of VIP^d^ > 1
**Plasma**	13	PLS-DA (mean-centered)	4	98.1%	0.827	R2 = (0.0, 0.753) Q2 = (0.0, -0.197)	63
**Males liver–aqueous**	15	PLS-DA (pareto scaled)^e^	1	77.2%	0.631	R2 = (0.0, 0.353) Q2 = (0.0, -0.17)	123
**Males liver–fatty acids**	20	OSC-mean centered + PLS-DA (mean-centered)	1	82.4%	0.805	R2 = (0.0, 0.037) Q2 = 0.0, -0.198)	3
**Females liver–aqueous**	20	PLS-DA (pareto scaled)	3	85.1%	0.474	R2 = (0.0, 0.461) Q2 = (0.0, -0.273)	78
**Females liver–fatty acids**	18	OSC-mean-centered + PLS-DA (pareto scaled)^f^	3	90.4%	0.754	R2 = (0.0, 0.467) Q2 = (0.0, -0.439)	5
**Males brain–aqueous**	17	OSC-mean centered + PLS-DA (mean centered)^g^	2	87.2%	0.668	R2 = (0.0, 0.364) Q2 = (0.0, -0.295)	48
**Females brain–aqueous**	20	PLS-DA (pareto scaled)	4	98.7%	0.783	R2 = (0.0, 0.837) Q2 = (0.0, -0.367)	147

^a^pool of three rats.

^b^ LV: latent variables.

^c^ 200 permutations.

^d^ VIP: variable importance in projection.

^e^ exclusion of rats n°G2-m-bis, G2-m-ter et G0-47.

^f^exclusion of rat n°G0-42.

^g^ exclusion of rat n°G0-43, G0-mter et G0-mbis.

**Table 7:** Metabolites for which the levels were significantly different between offspring from exposed and non-exposed dams.

Metabolite	Plasma ♂	Aqueous Liver ♂	Aqueous Liver ♀	Aqueous Brain ♂	Aqueous Brain ♀	Biological role
Creatine		↘	↘^a^^,^ ^b^			Amino-acid synthesis, storage energy
Lipids	↗					Lipid metabolism
ADP + AMP				↗		Energy source
Aspartate		↘				Non-essential amino-acid involved in urea cycle, DNA metabolism. Major excitatory neurotransmitter
Succinate				↗^b^		TCA cycle
Alanine		↘^b^		↘^b^		Energy source, glucose metabolism regulator
Glutamate	↘	↘		↘		Neoglucogenesis, excitatory neurotransmitter
Glutamine			↗	↘	↘	Non-essential amino-acid, role in TCA-cycle
Glucose		↗	↗			Energy source
Glycogen		↘	↘			Energy storage
Glycerol		↘	↘			Lipid component, converted to glucose for energy production
Lactate			↗^b^	↗^b^		Energy metabolism
Leucine		↘				Essential amino-acid involved in stress, energy and muscle metabolism, role in cholesterol synthesis
Lysine		↗^c^				Essential amino-acid involved in stress, precursor of acetyl-coA
Valine		↘				Branched-chain amino acid involved in stress, energy and muscle metabolism
Inosine					↗ ?	Purine nucleoside, intermediate in the degradation of purine nucleoside
Oxidized gluthatione		↘				Antioxidant
Taurine	↗^b^	↗	↗	↗		Membrane stabilizer, anti-oxidant, osmolyte
Glycerophosphocholine		↗^b^				Membrane stabilizer, osmolyte
Phosphocholine			↗			Membrane stabilizer, osmolyte
Choline	↘					Precursor of acetylcholine, lipid metabolism
Uridine					↗	Nucleoside, synthesis of RNA membrane, regulation of physiological processes

^a^ with creatinine.

^b^ tendency but without statistical significance (0,05<p<0,08).

^c^ with acetate.

#### Liver metabolic profiles

Aqueous extract: males and females were analyzed separately. Raw data are presented as bucket table in S6 and S7 Tables. For males, the preliminary PCA showed three outliers. After excluding them, the PCA correctly separate the exposed and control groups (Fig 2B). We found 13 metabolites with altered signals between the groups after PLS-DA modeling (Tables 6 and 7), including significant lower signals for creatine/ creatinine, glutamate, aspartate, glycogen, glycerol, leucine, valine, and glutathione, and higher signals for glucose, lysine (with acetate), taurine, and glycerophosphocholine for the male fetuses of exposed dams. Alanine was also decreased but in a non-significant way. This suggests a modification in glucose metabolism, potentially associated with mitochondrial dysfunction. Oxidative stress may also be possible for these changes. For females, the results were less informative, but there was a slight separation by PCA (Fig 2C) and the PLS-DA model explained more than 85% of the variability between groups (with a lower Q2 than for males, Table 7). The signals for only eight metabolites were different between groups, including decreases in glycogen, glycerol, and to a lesser extent creatine, and increases in glutamine, glucose, taurine, and phosphocholine, and to a lesser extent, lactate (Table 7).

Lipidic fraction: We specifically analyzed the fatty acids, as the livers of the male offspring of exposed dams were significantly heavier than those of control dams (Table 2). Males and females were analyzed separately. There was no statistical significant difference in the saturated/unsaturated fatty acid ratio between the exposed group and controls. Neither PCA nor PLS-DA separated the two groups. For males, an OSC filtered PLS-DA model allowed us to identify three fatty acids for which significant differences in the signals were confirmed using a Kruskal-Wallis test. The first component of the PLS-DA model explains 82.4% of the variability (Table 6). The signal for C16:0, C18:0 and C18:1ω9 in the exposed group was less than that of controls (Table 8). For females, no significant differences were confirmed after OSC filtered PLS-DA modeling that correctly separated the two groups (Table 6).

**Table 8:** Liver fatty acids for which the levels were significantly different between male offspring from the exposed and control groups (using the Kruskal Wallis test, p-value<0.05).

Fatty acids	Variables	Tendancy
Palmitic acid	OSC:C16:0	↘
Stearic acid	OSC:C18:0	↘
Oleic acid	OSC:C18:1ω9	↘

#### Brain metabolic profiles (aqueous fraction)

Males and females were analyzed separately. Raw data are presented as bucket table in S8 and S9 Tables. PLS-DA modelling allowed us to correctly separate the exposed and control groups for both sexes, but OSC-filtering was necessary for the males (Table 6). In males, lower signals were observed for glutamate and glutamine and higher signals for ADP/AMP and taurine. Alanine, lactate and succinate were also altered in a non-significant manner. In female, only two metabolites were modified, with an increase in uridine and a decrease in glutamine. These observations suggest a gender-specific sensitivity, with males possibly being more sensitive than females, as it was already suggested when comparing organ weights. For male, the observations may be consistent with those made in the dams that suggested a potential impact on amino-acid metabolism associated with oxidative stress.

## Discussion

Our results show that exposure during pregnancy to a low-dose of a mixture of eight pesticides, representative of human environmental exposure, may alter energy metabolism and the amino acid profile, as well as the metabolism of glucose and lipids, in both dams and offspring. The pesticide mixture was selected based on their use in Brittany, France, a region in which much of the surface area is devoted to cereal and corn crops. The eight pesticides (three herbicides, one fungicide, two insecticides, and two growth regulators) correspond to the most commonly used active substances in 2004, representing 90% of the pesticides used on cereal and corn crops.

The level of 36 endogenous metabolites were modified in the exposed group relative to the control group, both in dams and pups. Hippurate and phenylacetylglycine are involved in the metabolism of the gut microflora [39]. The changes observed in the excretion of these co-metabolites in dams (reduced urinary excretion of hippurate and phenylacetylglycine) may be associated with an impact on gut microflora function. The modification of these functions may be due to a direct effect, as shown by Zhao et al., after exposure of mice to chlorpyrifos [40]. However, it may also be related to increased intake of nitrogen compounds, inducing a concomitant enhancement of the urea cycle (increase in the excretion of urinary citrulline). Alanine is a non-essential amino acid that functions as a major energy source and regulator in glucose metabolism. Glutamate is a key amino acid in cellular metabolism. It is involved in glutathione metabolism and is also an excitatory neurotransmitter with a role in synaptic plasticity (associated with neurocognitive function). Glutamine is used to maintain the store of amino-acids in the organism and is formed from glutamate. The decrease in alanine levels (plasma levels in dams and liver and brain levels in pups) associated with decreased glutamate and glutamine levels suggest impairment of neoglucogenesis in the liver, associated with dysfunction of the TCA cycle. This hypothesis is strengthened by decrease in both glycogen and glycerol (precursors of glucose) levels and increased glucose levels observed in the liver of dams and pups. Furthermore, the slight increase in lactate in the livers and brains of pups supports the hypothesis of altered energy metabolism: enhancement of anaerobic metabolism is an alternative means to stimulate glycolysis by electron transfer, maintaining a steady state condition in the cell when the TCA cycle is perturbed [41]. These observations are consistent with altered glucose/energy metabolism in the liver. Such impairment has already been highlighted after exposure to propoxur alone [42,43] or an organophosphorus mixture [27].

Branched chain amino acids are the main nitrogen source for glutamine and alanine syntheses in the muscle. The decreased isoleucine and valine levels observed in the plasma of dams are consistent with the decreased alanine levels. These branched chain amino acids are involved in stress and energy metabolism and isoleucine also has an important role in fatty acid synthesis. The decrease observed in our study may be associated with the increased plasma lipid levels observed in all exposed animals relative to the control groups. Similar findings were reported for rodents orally exposed to single pesticides or pesticide mixtures: mice orally exposed to their respective ADI of endosulfan, atrazine, and chlorpyrifos [26], rats orally exposed to chlorpyrifos and carbaryl [29], propoxur alone [42,43], dimethoate alone [44], or a mixture of four organophosphorus pesticides [27].

We also propose enhancement of the beta-oxidation of fatty acids in response to the disruption of the TCA cycle, based on the increased levels of aceto-acetate in the liver in exposed dams, and the non-significant increases in 3-hydroxybutyrate level. This switch in energy metabolism from glycolysis to beta-oxidation was recently shown when rats were orally exposed to pyrethroids, and was associated with hepatotoxicity and nephrotoxicity [45]. This is supported by the decrease in urinary excretion of hippurate, which may be a marker of hepatic mitochondrial function [46], and phenylacetylglycine, which may indicate the disruption of FA metabolism. Indeed, decreased urinary excretion of hippurate was recently observed in rats exposed to acetochlor, the predominant pesticide included in the mixture studied (64%), and was associated with hepatic dysfunction [47]. In addition, the European Chemical Agency (ECHA) opinion on acetochlor [48] reported unpublished sub-chronic and chronic studies in dogs orally treated with acetochlor (doses between 2 and 75 mg/kg bw/d). These studies showed liver toxicity, resulting in an increase in relative liver weight and fat infiltration of the liver for some animals. These data suggest that the effect we observed in the liver could be driven by acetochlor, which was the predominant pesticide in our mixture. This is supported by recent findings published by Counihan *et al*. who showed that acetochlor could inhibit FA oxidation in mice, resulting in heightened levels of free FAs, triglycerides, cholesteryl esters, and other lipid species in the liver [49]. The alteration in FA profiles may be related to the inhibition of FA oxidation. Other pesticides, present in the mixture, have been found to alter lipid metabolism. Imidacloprid was reported to enhance adipogenesis *in vitro* and *in vivo* [50,51]. The herbicide Roundup, containing glyphosate, increased the accumulation of lipids in the liver of rats exposed to very low doses [52]. Glyphosate inhibited FA oxidation and increased fat and cholesteryl ester levels in the liver of mice [53]. These two chemicals may contribute to the effect we observed in the liver, especially in dams.

Glycine and glutathione are cytoprotective agents involved in ROS scavenging [54]. Taurine is a stabilizer of cell membranes. Glycerophosphocholine (GPC) is also an osmoprotective compound which has been found to be essential for the structural integrity of cell membranes [55]. Increased GPC concentrations to protect against cell damage can be induced by hypertonic conditions generated by oxidative stress, as shown in the case of liver damage [56]. Choline is a precursor of acetylcholine, as a methyl donor, in various metabolic processes, including lipid metabolism. Choline entering the cells is rapidly phosphorylated to phosphocholine or oxidized to betaine [57]. It is also involved in amino acid metabolism (glycine, serine, and threonine). The metabolic modifications observed in our study may be associated with impaired membrane function, associated with an oxidative stress, linked with mitochondrial dysfunction, especially in the liver, according to a previous study showing ROS generation after exposure to organophosphorous, organochlorine, or pyrethroid pesticides [58–60].

In brain, for male offspring, the decrease in alanine, glutamine, glutamate may be consistent with the alteration of energy metabolism and the TCA cycle. Oxidative stress can also be suggested, based on the increase in taurine levels in both dams and offspring (associated with an increase in oxidized glutathione in dams only). The decrease in ATP observed in dams could be a consequence of mitochondrial dysfunction. The inhibition of ATP synthesis has been experimentally observed after exposure to various organophosphorous insecticides, including monocrotophos, parathion, dichlorvos, metaphos, malathion and chlorpyrifos, as reviewed by Karami-Mohajeri and Abdollahi [61]. The depletion of intracellular ATP may be responsible for decreased activity of membrane channels (ATP-sensitive potassium channels), leading to membrane depolarization. The concomitant decrease in glutamate levels may lead to the opening of N-methyl-D-aspartate (NMDA) receptors, leading to altered neuron homeostasis (entry of calcium, apoptosis). Surprisingly, ATP is not modified in male offspring, and the modifications of ADP/ AMP levels were in the opposite direction. These observations need to be confirmed by other experimental studies to understand whether it is a direct effect associated with pesticide exposure or a compensatory mechanism resulting from the metabolic modifications observed in the mothers.

**Fig 3:**
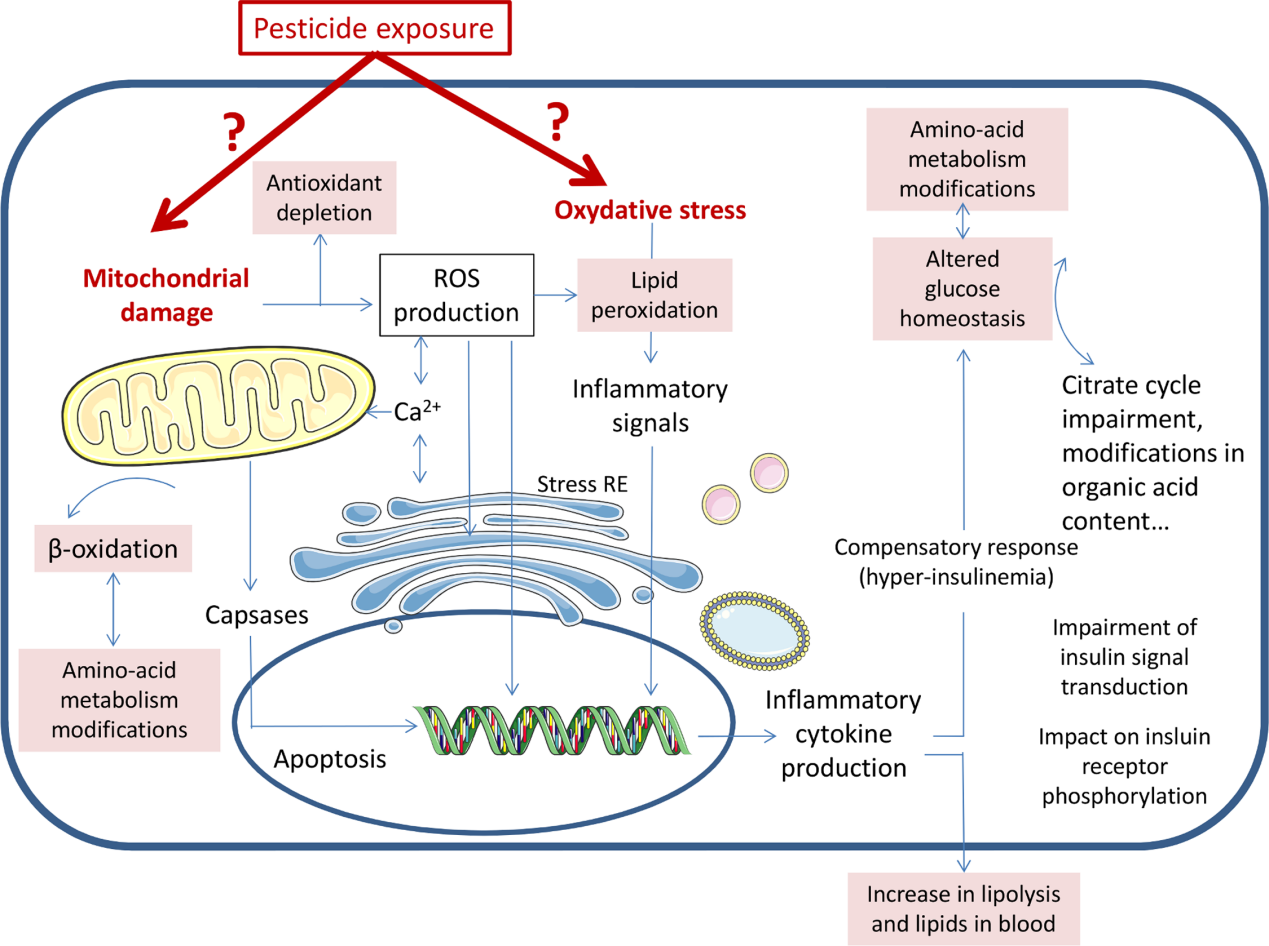
Suggestion of cellular mechanisms that may be induced by the exposure of pregnant rat to a mixture of eight pesticides (acetochlor, bromoxynil, carbofuran, chlormequat, ethephon, fenpropimorph, glyphosate, imidacloprid) from GD 4 to GD 21.

Finally, the most noteworthy differences in this study concern energy, amino acid, glucose, and lipid metabolism, potentially associated with liver dysfunction. Many metabolites are associated with the TCA cycle, such as citrate, succinate, lactate, glutamate, aspartate, and glutamine. The differences observed here may be linked to impaired neoglucogenesis (changes in alanine, glucose, glycogen, and glycerol), although there is no evidence as to whether it is a cause or a consequence of TCA cycle impairment. The levels of several osmoprotective metabolites were also modified (choline, phosphocholine, taurine, dimethylglycine, and glutathione), suggesting an adaptive response to oxidative stress. These observations suggest the cellular mechanism (Fig 3), in which oxidative stress and mitochondrial dysfunction may induce changes in cell signaling, with inflammatory phenomena as a potential cause of altered glucose homeostasis and increased plasma lipid levels. Mitochondrial dysfunction is largely mentioned and discussed in the current literature on insecticide exposure [61,62], whereas liver dysfunction appears to be linked mostly with exposure to fungicides (primarily conazoles) and herbicides [63]. However, the latest evidence also supports an association between liver dysfunction and organophosphorous insecticides [30,64,65].

In conclusion, these observations suggest that maternal exposure to a mixture of eight pesticides commonly used in agriculture gives rise to oxygen containing free radicals, and the production of ROS, which may induce stress in the endoplasmic reticulum, leading to an inflammatory process responsible for a compensatory response through glucose and lipid metabolism. The analyses of liver tissue extracts suggest altered regulation of fatty acid composition and lipid storage in dams and male offspring. Modifications observed in offspring may be a consequence of the altered metabolism in dams. The analyses of brain tissue extracts suggest an impact on energy metabolism, both in dams and male pups. Finally, our observations are relatively similar to other published studies, which have investigated the exposure of various types of pesticides (organophosphorous, carbamates, organochlorines, pyrethroids, alone or in combination) and are consistent with the metabolomics data from pregnant women in Brittany exposed to pesticides [33]. These observations may favor a unique signature that characterizes pesticide exposure, accounting for a common adaptive response by exposed animals. It is not possible to draw a clear conclusion based on the analyses of the dams, but it cannot be excluded that the metabolic modifications observed in pups, in both the liver and brain, especially for males, may have an adverse impact later in life (metabolic disorders, neurological effects).

## Supporting information

S1 Table Bucket Table corresponding to NMR spectra from urine of dams collected at GD15. (XLSX)

S2 Table Bucket Table corresponding to NMR spectra from plasma of dams collected at GD21. (XLSX)

S3 Table Bucket Table corresponding to NMR spectra from aqueous liver extracts of dams collected at GD21. (XLSX)

S4 Table Bucket Table corresponding to NMR spectra from aqueous brain extracts of dams collected at GD21. (XLSX)

S5 Table Bucket Table corresponding to NMR spectra from plasma of male offspring collected at GD21. (XLSX)

S6 Table Bucket Table corresponding to NMR spectra from aqueous liver extract of male offspring collected at GD21. (XLSX)

S7 Table Bucket Table corresponding to NMR spectra from aqueous liver extract of female offspring collected at GD21. (XLSX)

S8 Table Bucket Table corresponding to NMR spectra from aqueous brain extract of male offspring collected at GD21. (XLSX)

S9 Table Bucket Table corresponding to NMR spectra from aqueous brain extract of female offspring collected at GD21. (XLSX)

## References

[1] Bradman A, Castorina R, Boyd Barr D, Chevrier J, Harnly ME, Eisen EA et al Determinants of organophosphorus pesticide urinary metabolite levels in young children living in an agricultural community. International Journal of Environmental Research and Public Health. 2011; 8: 1061–1083. doi: 10.3390/ijerph8041061 21695029 10.3390/ijerph8041061 PMC3118878

[2] Chevrier C, Serrano T, Lecerf R, Limon G, Petit C, Monfort C et al Environmental determinants of the urinary concentrations of herbicides during pregnancy: the PELAGIE mother-child cohort (France). Environment International. 2014; 63: 11–18. doi: 10.1016/j.envint.2013.10.010 24246238 10.1016/j.envint.2013.10.010

[3] Gunier RB, Ward MH, Airola M, Bell EM, Colt J, Nishioka M et al Determinants of agricultural pesticide concentrations in carpet dust. Environmental Health Perspectives. 2011; 19: 970–976. 10.1289/ehp.1002532 PMC3222988 21330232

[4] Jamin E, Bonvallot N, Tremblay-Franco M, Cravedi JP, Chevrier C, Cordier S et al Untargeted profiling of pesticide metabolites by LC-HRMS: an exposomics tool for human exposure evaluation. Analytical and Bioanalytical Chemistry. 2014; 406: 1149–1161. doi: 10.1007/s00216-013-7136-2 23892877 10.1007/s00216-013-7136-2

[5] Ward MH, Lubin J, Giglierano J, Colt JS, Wolter C, Bekiroglu N et al Proximity to crops and residential exposure to agricultural herbicides in Iowa. Environmental Health Perspectives. 2006; 114(6): 893–897. doi: 10.1289/ehp.8770 16759991 10.1289/ehp.8770 PMC1480526

[6] Alavanja M, Hoppin JA, Kamel F. Health effects of chronic pesticide exposure: cancer and neurotoxicity. Annual Review in Public health. 2004; 25: 155–197. 10.1146/annurev.publhealth.25.101802.123020 15015917

[7] Hanke W and Jurewicz J. The risk of adverse reproductive and developmental disorders due to occupational pesticide exposure: an overview of current epidemiological evidence. International Journal of Occupational Medicine and Environmental Health. 2004; 17(2): 223–243. 15387079

[8] Mattix KD, Winchester PD, Scherer LR. Incidence of abdominal wall defects is related to surface water atrazine and nitrate levels. Journal of Pediatric Surgery. 2007; 42: 947–949. doi: 10.1016/j.jpedsurg.2007.01.027 17560200 10.1016/j.jpedsurg.2007.01.027

[9] Waller SA, Paul K, Peterson SE, Hitti JE. Agricultural-related chemical exposures, season of conception, and risk of gastroschisis in Washington State. American Journal of Obstetrics & Gynecology. 2010; 202: 241–246. 20207240 10.1016/j.ajog.2010.01.023

[10] Weselak M, Arbuckle TE, Wigle DT, Walker MC, Krewski D. Pre- and post-conception pesticide exposure and the risk of birth defects in an Ontario farm population. Reproductive Toxicology. 2008; 25: 472–480. doi: 10.1016/j.reprotox.2008.05.060 18586452 10.1016/j.reprotox.2008.05.060

[11] Chevrier C, Limon G, Monfort C, Rouget F, Garlantezec R, Petit C et al Urinary biomarkers of prenatal atrazine exposure and adverse birth outcomes in the PELAGIE birth cohort. Environmental Health Perspective. 2011; 119: 1034–1041. 10.1289/ehp.1002775 PMC3222984 21367690

[12] Hochoa-Acuna H, Frankenberger J, Leighanne H, Carbajo C. Drinking-water herbicide exposure in Indiana and prevalence of small-for-gestational-age and preterm delivery. Environmental Health Perspectives. 2009; 117: 1619–1624. doi: 10.1289/ehp.0900784 20019915 10.1289/ehp.0900784 PMC2790519

[13] Harley KG, Huen K, Aguilar SR, Holland NT, Bradman A, Barr DB et al Association of organophosphate pesticide exposure and paraoxonase with birth outcome in Mexican-American women. PLoS One. 2011; 6: e23923 doi: 10.1371/journal.pone.0023923 21904599 10.1371/journal.pone.0023923 PMC3164135

[14] Eskenazi B, Huen K, Marks A, Harley KG, Bradman A, Barr DB et al PON1 and neurodevelopment in children from the CHAMACOS study exposed to organophosphate pesticides in utero. Environmental Health Perspectives. 2010; 118: 1775–1781. doi: 10.1289/ehp.1002234 21126941 10.1289/ehp.1002234 PMC3002199

[15] Marks AR, Harley K, Bradman A, Kogut K, Barr DB, Johnson C et al Organophosphate pesticide exposure and attention in young Mexican-American children: the CHAMACOS study. Environmental Health Perspectives. 2010; 118(12): 1768–1774. doi: 10.1289/ehp.1002056 21126939 10.1289/ehp.1002056 PMC3002198

[16] Ostrea EM JR., Reyes A, Villanueva-Uy E, Pacifico R, Benitez B, Ramos E et al Fetal exposure to propoxur and abnormal child neurodevelopment at 2 years of age. Neurotoxicology. 2012; 33: 669–675. doi: 10.1016/j.neuro.2011.11.006 22155319 10.1016/j.neuro.2011.11.006 PMC3509383

[17] Rauh VA, Garfinkel R, Perera FP, Andrews HF, Hoepner L, Barr DB et al Impact of prenatal chlorpyrifos exposure on neurodevelopment in the first 3 years of life among inner-city children. Pediatrics. 2006; 118: e1845–e1859. doi: 10.1542/peds.2006-0338 17116700 10.1542/peds.2006-0338 PMC3390915

[18] Rauh V, Arunajadai S, Horton M, Perera F, Hoepner L, Barr DB et al Seven-year neurodevelopmental scores and prenatal exposure to chlorpyrifos, a common agricultural pesticide. Environmental Health Perspectives. 2011; 119: 1196–1201. doi: 10.1289/ehp.1003160 21507777 10.1289/ehp.1003160 PMC3237355

[19] Quirós-Alcalá L, Mehya S, Eskenazi B. Pyrethroid pesticide exposure and parental report of learning disability and attention deficit/hyperactivity disorder in U.S. children: NHANES 1999–2002. Environmental Health Perspectives. 2014; 122(12): 1336–1342. doi: 10.1289/ehp.1308031 25192380 10.1289/ehp.1308031 PMC4256700

[20] Viel JF, Warembourg C, Le Maner-Idrissi G, Lacroix A, Limon G, Rouget F et al Pyrethroid insecticide exposure and cognitive developmental disabilities in children: The PELAGIE mother-child cohort. Environment International. 2015; 82: 69–75. doi: 10.1016/j.envint.2015.05.009 26057254 10.1016/j.envint.2015.05.009

[21] Viel JF, Rouget F, Warembourg C, Monfort C, Limon G, Cordier S et al Behavioural disorders in 6-year-old children and pyrethroid insecticide exposure: the PELAGIE mother–child cohort. Occupational and Environmental Medicine. 2017; 74: 275–281. doi: 10.1136/oemed-2016-104035 28250046 10.1136/oemed-2016-104035

[22] Wagner-Schuman M, Richardson JR, Auinger P, Braun JM, Lanphear BP, Epstein JN et al Association of pyrethroid pesticide exposure with attention-deficit/hyperactivity disorder in a nationally representative sample of U.S. children. Environmental Health. 2015; 14: 44–52. doi: 10.1186/s12940-015-0030-y 26017680 10.1186/s12940-015-0030-y PMC4458051

[23] Watkins DJ, Fortenberry GZ, Sanchez BN, Barr DB, Panuwet P, Schnaas L et al Urinary 3-phenoxybenzoic acid (3-PBA) levels among pregnant women in Mexico City: distribution and relationships with child neurodevelopment. Environmental Research. 2016; 147: 307–313. doi: 10.1016/j.envres.2016.02.025 26922411 10.1016/j.envres.2016.02.025 PMC4821665

[24] Bonvallot N, Tremblay-Franco M, Chevrier C, Canlet C, Debrauwer L, Cravedi JP et al Potential input from metabolomics for exploring and understanding the links between environment and health. Journal of Toxicology and Environmental Health, Part B: Critical Reviews. 2014; 17(1): 21–44. 10.1080/10937404.2013.860318 24597908

[25] Canlet C, Tremblay-Franco M, Gautier R, Molina J, Métais B, Blas-Y-Estrada F et al Specific metabolic fingerprint of a dietary exposure to a very low dose of endosulfan. Journal of Toxicology. 2013; 545802 doi: 10.1155/2013/545802 23431292 10.1155/2013/545802 PMC3569910

[26] Demur C, Métais B, Canlet C, Tremblay-Franco M, Gautier R, Blas-Y-Estrada F et al Dietary exposure to a low dose of pesticides alone or as a mixture: the biological metabolic fingerprint and impact on hematopoiesis. Toxicology. 2013; 308: 74–87. doi: 10.1016/j.tox.2013.03.004 23528616 10.1016/j.tox.2013.03.004

[27] Du L, Wang H, Xu W, Zeng Y, Hou Y, Zhang Y et al Application of ultraperformance liquid chromatography/mass spectrometry-based metabonomic techniques to analyze the joint toxic action of long-term low-level exposure to a mixture of organophosphate pesticides on rat urine profile. Toxicological Sciences. 2013; 134(1): 195–206. doi: 10.1093/toxsci/kft091 23580312 10.1093/toxsci/kft091

[28] Wang HP, Liang YJ, Long DX, Chen JX, Hou WY, Wu YJ. Metabolic profiles of serum from rats after subchronic exposure to chlorpyrifos and carbaryl. Chemical Research in Toxicology. 2009; 22: 1026–1033. doi: 10.1021/tx8004746 19522548 10.1021/tx8004746

[29] Wang HP, Liang YJ, Zhang Q, Long DX, Li W, Li L et al Changes in metabolic profiles of urine from rats following chronic exposure to anticholinesterase pesticides. Pesticide Biochemistry and Physiology. 2011; 101: 232–239.

[30] Cao C, Zeng Y, Shi H, Yang S, Bao W, Qi L et al Metabonomic analysis of quercentin against the toxicity of chronic exposure to a mixture of four organophosphate pesticides in rat plasma. Xenobiotica. 2016; 46(9): 805–815. doi: 10.3109/00498254.2015.1121552 26677787 10.3109/00498254.2015.1121552

[31] Wang HP, Liang YJ, Sun YJ, Chen JX, Hou WY, Long DX et al 1H NMR-based metabonomic analysis of the serum and urine of rats following subchronic exposure to dichlorvos, deltamethrin, or a combination of these two pesticides. Chemico-Biological Interactions. 2013; 203: 588–596. doi: 10.1016/j.cbi.2013.03.017 23566885 10.1016/j.cbi.2013.03.017

[32] Merhi M, Demur C, Racaud-Sultan C, Bertrand J, Canlet C, Blas-Y-Estrada F et al Gender-linked haematopoietic and metabolic disturbances induced by a pesticide mixture administered at low dose to mice. Toxicology. 2010; 267: 80–90. doi: 10.1016/j.tox.2009.10.024 19883720 10.1016/j.tox.2009.10.024

[33] Bonvallot N, Tremblay-Franco M, Chevrier C, Canlet C, Warembourg C, Cravedi JP et al Metabolomics tools for describing complex pesticide exposure in pregnant women in Brittany (France). PLoS One. 2013; 8(5): e64433 doi: 10.1371/journal.pone.0064433 23704985 10.1371/journal.pone.0064433 PMC3660334

[34] Waters NJ, Holmes E, Waterfield CJ, Farrant RD, Nicholson JK. NMR and pattern recognition studies on liver extracts and intact livers from rats treated with alpha-naphthylisothiocyanate. Biochemical Pharmacology. 2002; 64: 67–77. 12106607 10.1016/s0006-2952(02)01016-x

[35] Eriksson L, Johansson E, Kettaneh-Wold N, Trygg J, Wikstrom C, Wold S. Multi- and megavariate data analysis Part I Basic principles and applications. 2006 Second revised and enlarged edition. Umetrics Academy.

[36] Wold S, Antti H, Lindgren F, Ohman J. Orthogonal signal correction of near-infrared spectra. Chemometrics and Intelligent Laboratory Systems. 1998; 44: 175–185.

[37] Benjamini Y and Hochberg Y. Controlling the false discovery rate: a practical and powerful approach to multiple testing. Journal of the Royal Statistical Society, Series B. 1995: 57(1): 289–300.

[38] Al-Eryani L, Wahlang B, Falkner KC, Guardiola JJ, Clair HB, Prough RA et al Identification of environmental chemicals associated with the development of toxicant-associated fatty liver disease in rodents. Toxicology Pathology. 2015; 43(4): 482–497. 10.1177/0192623314549960 PMC4501484 25326588

[39] Nicholson JK, Holmes E, Wilson ID. Gut microorganisms, mammalian metabolism and personalized health care. Nature Reviews Microbiology. 2005; 3: 431–438. doi: 10.1038/nrmicro1152 15821725 10.1038/nrmicro1152

[40] Zhao Y, Zhang Y, Wang G, Han R, Xie X. Effects of chlorpyrifos on the gut microbiome and urine metabolome in mouse (Mus musculus). Chemosphere. 2016; 153: 287–93. doi: 10.1016/j.chemosphere.2016.03.055 27018521 10.1016/j.chemosphere.2016.03.055

[41] Verwaest KA, Vu TN, Laukens K, Clemens LE, Nguyen HP, Van Gasse B et al 1H NMR based metabolomics of CSF and blood serum: a metabolic profile for a transgenic rat model of Huntington disease. Biochimica et Biophysica Acta (BBA)—Molecular Basis of Disease. 2011; 1812: 1371–1379. 21867751 10.1016/j.bbadis.2011.08.001

[42] Liang YJ, Wang HP, Long DX, Wu YJ. (1)H NMR-based metabonomic profiling of rat serum and urine to characterize the subacute effects of carbamate insecticide propoxur. Biomarkers. 2012; 17: 566–574. doi: 10.3109/1354750X.2012.704527 22780197 10.3109/1354750X.2012.704527

[43] Liang YJ, Wang HP, Long DX, Wu YJ. Applying biofluid metabonomic techniques to analyze the combined subchronic toxicity of propoxur and permethrin in rats. Bioanalysis. 2012; 4: 2897–2907. doi: 10.4155/bio.12.277 23244281 10.4155/bio.12.277

[44] Feng Z, Sun X, Yang J, Hao D, Du L, Wang H et al Metabonomics analysis of urine and plasma from rats given long-term and low-dose dimethoate by ultra-performance liquid chromatography-mass spectrometry. Chemico-Biological Interactions. 2012; 199: 143–153. doi: 10.1016/j.cbi.2012.07.004 22884955 10.1016/j.cbi.2012.07.004

[45] Liang YJ, Wang HP, Long DX, Li W, Wu YJ. A metabonomic investigation of the effects of 60 days exposure of rats to two types of pyrethroid insecticides. Chemico-Biological Interactions. 2013; 206(2): 302–308. doi: 10.1016/j.cbi.2013.10.002 24121187 10.1016/j.cbi.2013.10.002

[46] Krähenbül L, Reichen J, Talos C, Krähenbül S. Benzoic acid metabolism reflects hepatic mitochondrial function in rats with long-term extrahepatic cholestasis. Hepatology. 1997; 25(2): 278–283. doi: 10.1053/jhep.1997.v25.pm0009021934 9021934 10.1053/jhep.1997.v25.pm0009021934

[47] Li L, Wang M, Chen S, Zhao W, Zhao Y, Wang X et al A urinary metabonomics analysis of long-term effect of acetochlor exposure on rats by ultra-performance liquid chromatography/ mass spectrometry. Pesticide Biochemistry and Physiology. 2016; 128: 82–88. doi: 10.1016/j.pestbp.2015.09.013 26969444 10.1016/j.pestbp.2015.09.013

[48] ECHA. Committee for risk assessment RAC. Opinion proposing harmonised classification and labelling at EU level of acetochlor (ISO); 2-chloro-N-(ethoxymethyl)-N-(2-ethyl-6-methylphenyl)acetamide. Adopted 04 December 2014.

[49] Counihan JL, Duckering M, Dalvie E, Ku WM, Bateman LA, Fisher KJ et al Chemoproteomic profiling of acetanilide herbicides reveals their role in inhibiting fatty acid oxidation. ACS Chemical Biology. 2017; 12(3): 635–642. doi: 10.1021/acschembio.6b01001 28094496 10.1021/acschembio.6b01001 PMC6376971

[50] Park Y, Kim Y, Kim J, Yoon KS, Clark J, Lee J et al Imidacloprid, a neonicotinoid insecticide, potentiates adipogenesis in 3T3-L1 adipocytes. Journal of Agricultural and Food Chemistry. 2013; 61: 255–259. doi: 10.1021/jf3039814 23215241 10.1021/jf3039814

[51] Sun Q, Xiao X, Kim Y, Kim D, Yoon KS, Clark JM et al Imidacloprid promotes high fat diet-induced adiposity and insulin resistance in male C57BL/6J mice. Journal of Agricultural and Food Chemistry. 2016; 64: 9293–9306. doi: 10.1021/acs.jafc.6b04322 27960282 10.1021/acs.jafc.6b04322 PMC5325319

[52] Mesnage R, Renney G, Seralini GE, Ward M, Antoniou MN. Multiomics reveal non-alcoholic fatty liver disease in rats following chronic exposure to an ultra-low dose of Roundup herbicide. Nature Scientific Reports. 2017; 7: 39328. 10.1038/srep39328 PMC5220358 28067231

[53] Ford B, Bateman LA, Gutierrez-Palominos L, Park R, Nomura DK. Mapping Proteome-wide targets of glyphosate in mice. Cell Chemical Biology. 2017; 24: 1–8. doi: 10.1016/j.chembiol.2017.01.001 28132892 10.1016/j.chembiol.2016.12.013

[54] Petrat F, Boengler K, Schulz R, de Groot H. Glycine, a simple physiological compound protecting by yet puzzling mechanism(s) against ischaemia-reperfusion injury: current knowledge. British Journal of Pharmacology. 2012; 165: 2059–2072. doi: 10.1111/j.1476-5381.2011.01711.x 22044190 10.1111/j.1476-5381.2011.01711.x PMC3413844

[55] Klein J. Membrane breakdown in acute and chronic neurodegeneration: focus on choline-containing phospholipids. Journal of Neural Transmission. 2000; 107: 1027–1063. doi: 10.1007/s007020070051 11041281 10.1007/s007020070051

[56] Zhang L, Ye Y, An Y, Tian Y, Wang Y, Tang H. Systems responses of rats to aflatoxin B1 exposure revealed with metabonomic changes in multiple biological matrices. Journal of Proteome Research. 2011; 10: 614–623. doi: 10.1021/pr100792q 21080729 10.1021/pr100792q

[57] Pritchard PH, Vance DE. Choline metabolism and phosphatidylcholine biosynthesis in cultured rat hepatocytes. Biochemical Journal. 1981; 196: 261–267. 6272753 10.1042/bj1960261 PMC1162990

[58] Lee JE, Park JH, Shin IC, Koh HC. Reactive oxygen species regulated mitochondria-mediated apoptosis in PC12 cells exposed to chlorpyrifos. Toxicology and Applied Pharmacology. 2012; 263(2): 148–162. doi: 10.1016/j.taap.2012.06.005 22714038 10.1016/j.taap.2012.06.005

[59] Li HY, Wu SY, Ma Q, Shi N. The pesticide deltamethrin increases free radical production and promotes nuclear translocation of the stress response transcription factor Nrf2 in rat brain. Toxicology and Industrial Health. 2011; 27: 579–590. doi: 10.1177/0748233710393400 21398409 10.1177/0748233710393400 PMC4658656

[60] Pathak R, Mustafa MD, Ahmed T, Ahmed R, Tripathi AK, Guleria K et al Intra uterine growth retardation: Association with organochlorine pesticide residue levels and oxidative stress markers. Reproductive Toxicology. 2011; 31: 534–539. doi: 10.1016/j.reprotox.2011.02.007 21338667 10.1016/j.reprotox.2011.02.007

[61] Karami-Mohajeri S, Abdollahi M. Mitochondrial dysfunction and organophosphorus compounds. Toxicology and Applied Pharmacology. 2013; 270: 39–44. doi: 10.1016/j.taap.2013.04.001 23578477 10.1016/j.taap.2013.04.001

[62] Baltazar MT, Dinis-Oliveira RJ, de Lourdes Bastos M, Tsatsakis AM, Duarte JA, Carvalho F. Pesticides exposure as etiological factors of Parkinson's disease and other neurodegenerative diseases—a mechanistic approach. Toxicology Letters. 2014; 230(2): 85–103. doi: 10.1016/j.toxlet.2014.01.039 24503016 10.1016/j.toxlet.2014.01.039

[63] Al-Eryani L, Wahlang B, Falkner KC, Guardiola JJ, Clair HB, Prough RA et al Identification of environmental chemicals associated with the development of toxicant-associated fatty liver disease in rodents. Toxicology Pathology. 2015; 43(4): 482–497. 10.1177/0192623314549960 PMC4501484 25326588

[64] Deng Y, Zhang Y, Lu Y, Zhao Y, Ren H. Hepatotoxicity and nephrotoxicity induced by the chlorpyrifos and chlorpyrifos-methyl metabolite, 3,5,6-trichloro-2-pyridinol, in orally exposed mice. Science of the Total Environment. 2016; 544: 507–514. doi: 10.1016/j.scitotenv.2015.11.162 26674679 10.1016/j.scitotenv.2015.11.162

[65] Hou Y, Cao C, Bao W, Yang S, Shi H, Hao D et al The plasma metabolic profiling of chronic acephate exposure in rats via an ultra-performance liquid chromatography-mass spectrometry based metabonomic method. Molecular Biosystems. 2015; 11(2): 506–515. doi: 10.1039/c4mb00523f 25418677 10.1039/c4mb00523f

